# Association Between Adiposity Rebound and the Frequency of Balanced Meals Among Japanese Preschool Children: A Cross-Sectional Study

**DOI:** 10.3390/nu17193183

**Published:** 2025-10-09

**Authors:** Yuki Tada, Kemal Sasaki, Tomomi Kobayashi, Yasuyo Wada, Daisuke Fujita, Tetsuji Yokoyama

**Affiliations:** 1 Department of Nutritional Science, Tokyo University of Agriculture, Tokyo 156-8502, Japan; 2 Department of Food and Health Sciences, Jissen Women’s University, Tokyo 191-8510, Japan; sasaki-kemal@jissen.ac.jp; 3 Department of Food Science and Nutrition, Mukogawa Women’s University, Nishinomiya 663-8558, Japan; t_koba@mukogawa-u.ac.jp; 4 Department of Health Promotion, National Institute of Public Health, Wako 351-0197, Japan; wada.y.aa@niph.go.jp (Y.W.); yokoyama.t.aa@niph.go.jp (T.Y.); 5 Department of Food and Nutritional Science, Graduate School of Applied Bioscience, Tokyo University of Agriculture, Tokyo 156-8502, Japan; 11324001@nodai.ac.jp

**Keywords:** adiposity rebound, preschool child, dietary intake, Japanese

## Abstract

Background: The Healthy Japan 21-Phase III dietary recommendations comprise a staple food, main dish, and side dish to maintain nutritional balance and support healthy child growth. The relationship between the frequency of such balanced meals and early adiposity rebound (AR), a predictor of obesity, remains unclear. Objective: This study aimed to examine the association between the frequency of balanced meals (staple food, main dish, and side dish) and early AR in preschool children. Methods: In this cross-sectional secondary analysis of nationwide online survey data of 688 mothers of children aged 3–6 years, dietary habits were assessed using a validated NutriSTEP-based 22-item Japanese Nutrition Screening Questionnaire. AR constituted a body mass index (BMI) increase from the 18- to 36-month health checkups recorded in the Maternal and Child Health Handbook. Risk scores reflecting lower frequency of balanced meals were calculated for staple foods, main dishes, and side dishes. Logistic regression evaluated associations between dietary risk scores and AR, adjusting for the child’s sex, age, gestational age, birth weight, daycare attendance, and parental obesity. Results: Among 688 children, 193 (28.1%) exhibited early AR and had significantly higher BMI at age 3 and the most recent measurement (both *p* < 0.01). A higher total dietary risk score was independently associated with AR (adjusted odds ratio; 2.58 [95% CI: 1.08–6.16]). In addition, the absolute risk difference between high- and low-risk groups was 8.5% (95% CI: 1.7–15.2%). Conclusions: A lower frequency of balanced meals is associated with early AR. These findings suggest that a simple, meal-balance screening tool could potentially aid in the early identification of the risk of later obesity and timely nutritional guidance.

## 1. Introduction

In early childhood, the nutritional status is influenced by various factors, such as energy and nutrient intake and lifestyle habits. A high body mass index (BMI) in childhood is associated with various diseases, including obesity, diabetes, and cancer, in adulthood [1]. Worldwide, in 2022, approximately 159 million children were classified as obese; considering the 880 million obese adults in that year, the total number of obese individuals exceeded one billion [2]. To reduce obesity rates, it is crucial to monitor the nutritional status from early childhood and establish favorable eating habits to prevent obesity.

Indicators of nutritional status in children include the BMI for age z-score (BAZ) and weight-for-age z-score (WAZ), both of which are part of the World Health Organization (WHO) growth standards and have been widely used as public health indicators [3]. Adiposity Rebound (AR) indicates intraindividual changes in fat accumulation. In longitudinal studies, children with AR and an increase in BMI during early childhood had a higher risk of future obesity and metabolic disorders [4,5,6,7]. Recent nationwide data from Korea indicated that early AR can be influenced by infant-feeding patterns and the intake of sugar-sweetened beverages [8]. Therefore, AR monitoring is an effective method for the early identification of children at risk of obesity and metabolic abnormalities. The identification of AR-associated dietary factors could enable the implementation of preventive measures against lifestyle diseases in early childhood. Excessive energy intake during early childhood and high maternal BMI are AR-associated factors [9]. A high maternal BMI is linked to unhealthy dietary patterns in children during early childhood [10]. In particular, a poor-quality diet in early childhood hinders the improvement of diet quality during the growth phase [11]; moreover, weight-loss interventions that are implemented after the onset of obesity have limited effectiveness [12]. Therefore, ensuring a nutritionally balanced diet from early childhood is essential for supporting the healthy growth and development of young children and plays a crucial role in suppressing excessive fat accumulation and promoting healthy growth.

The National Health Promotion Initiative in Japan (Healthy Japan 21, Phase III) recommends a well-balanced diet, which includes staple foods as well as main and side dishes [13]. Staple foods comprise carbohydrate-rich foods, such as rice, bread, and noodles; the main dish includes protein-rich foods, such as meat, fish, eggs, and soy products; and the side dish consists of vegetables, mushrooms, seaweed, and root vegetables. A study on Japanese adults revealed that a higher frequency consumption of meals comprising a staple food, main dish, and side dish was associated with a greater intake of energy, protein, vitamins, and minerals and a higher probability of meeting the Japanese dietary intake standards [14]. Furthermore, high adherence to the dietary balance guide, which incorporates staple foods, main dishes, side dishes, dairy products, fruits, and snacks/beverages [15], reduced all-cause and cardiovascular mortality risks in middle-aged and older men and women [16,17]. Therefore, the recommended dietary pattern is important for maintaining nutritional balance and is expected to contribute significantly to the healthy growth of young children.

Nonetheless, the proportion of young children who incorporate staple foods, main dishes, and side dishes into their meals remains unclear, and the relationship between this dietary pattern and body composition remains unelucidated. Recent analyses of food-consumption trends in Japanese children reported declining intakes of fish, dairy products, and fruits, and thereby highlight the need for early dietary interventions [18]. Data from the National Health and Nutrition Survey in 2023 on the proportion of individuals aged ≥20 years who consume a meal comprising a staple food, main dish, and side dish at least twice a day showed that only 28–40% of men and women in their 30s and 40s—who constitute the child-rearing generation—met this criterion, which implies that their children may also have insufficient dietary quality. Thus, if consuming meals with a staple food, main dish, and side dish is associated with children’s future obesity and health, then the strong correlation of familial dietary habits [19] underscores the relevance of improved eating habits of the entire family. In recent years, reports have emphasized the importance of family meal frequency and shared eating habits in preventing childhood overweight and obesity [20]. Thus, the definitive association of AR with the scores of a simple dietary balance assessment tool for monitoring the intake of staple food, main dishes, and side dishes could facilitate its application for rapid screening during infant health checkups.

The present analysis aimed to clarify the association between the frequency of balanced meals—defined as meals including a staple food, a main dish, and a side dish—and early AR among Japanese preschool children. Beyond clarifying this relationship, we highlight the novelty of our approach: the frequency of balanced meals may serve as a simple, actionable screening indicator that can be readily incorporated into routine pediatric health checkups. Such an approach has the potential to provide healthcare professionals with a feasible tool for early risk identification and to lay the groundwork for future prospective studies aimed at confirming these associations.

## 2. Materials and Methods

A self-administered online questionnaire survey that targeted the mothers of children aged 18 months to 6 years (n = 1500) was conducted as part of the “Statistical Analysis of the Survey on Physical Growth of Preschool and Research on the Evaluation of Growth, Development, and Nutritional Status in Preschool Children” research project that was funded by the 2024 Fiscal Year Child and Family Administration Promotion Research Grant. This study involves a secondary subcohort analysis of the survey data obtained from respondents who were mothers to children aged 3–6 years (n = 750). As this research involves a secondary analysis, no formal sample-size calculation was performed.

### 2.1. Participants and Survey

#### 2.1.1. Participants

The participants of the original study were mothers registered with NTT Com Online Marketing Solutions Corporation (Shinagawa, Tokyo, Japan) (https://www.nttcoms.com/en/about/outline/ (accessed on 14 August 2025)) who were living with children aged 18 months to 6 years. The inclusion criteria were residence in Japan and Japanese as the mother tongue. Multiparas and healthcare professionals (i.e., physicians, dentists, pharmacists, nurses, public health nurses, midwives, clinical laboratory technicians, dietitians, registered dietitians, physical therapists, occupational therapists, and speech-language therapists) were excluded from the study. To ensure a representative sample, based on the 2020 Census data on the proportion of general households with children under 6 years, we defined 12 regional blocks within Japan, whereby the number of participants from each block was determined proportionally, and only respondents within the target cutoff for the respective block were included. The survey was conducted from 27 May to 6 June 2024, when the target sample size was reached.

#### 2.1.2. Survey

Data on participant demographics included the child’s sex, age, daycare use, birth weight, and gestational age, as well as the parents’ height, weight, and employment status. Moreover, information on the child’s height and weight at 18 and 36 months as well as at the most recent measurement, was recorded. In Japan, the Maternal and Child Health Handbook constitutes a record of the health status of mothers and children throughout pregnancy, childbirth, and child-rearing. This handbook contains records of pregnancy and childbirth, mandatory health checkups for children up to elementary school entry (at 18 and 36 months), immunizations, and dental checkups. To ensure accurate reporting of children’s physical measurements, mothers were asked to provide photographs of height and weight measurements undertaken during the mandatory health checkups at 18 and 36 months that were recorded in the Maternal and Child Health Handbook.

To assess dietary habits, we used a provisional 22-item Japanese version of the Nutrition Screening Questionnaire [21], which was developed with reference to the Nutrition Screening Tool for Every Preschooler (NutriSTEP) [22] that was originally designed in Canada to evaluate the nutritional status and dietary habits of preschoolers. The Japanese version of the questionnaire was created based on literature reviews, national infant nutrition surveys, infant health checkups, surveys on changes in lifestyle owing to the coronavirus disease (COVID-19) pandemic, and municipal survey results, further adapted to reflect Japan’s unique dietary culture and eating habits [21] and was designed for parents who were familiar with the child’s eating habits, with mothers responding on behalf of their children in this study. The questionnaire consisted of 22 items: (1) food group intake frequency (8 items: cereals, dairy products, vegetables, fruits, seafood, meat, eggs, and soybeans/soy products); (2) undesirable food intake frequency (3 items: fast food, snacks/sugary processed sweets, and sweetened beverages); (3) eating habits (6 items: parental concerns about meals, parental concerns about chewing, feeling hungry before meals, meal intake frequency, eating while doing something else, and frequency of eating together); (4) other lifestyle habits (2 items: physical activity and smartphone/tablet usage); (5) parental perception of child’s weight (1 item); (6) parental time flexibility (1 item); and (7) financial stability (1 item).

### 2.2. Validation and Reliability of the Survey

To logical validity of the questionnaire was assessed based on evaluations by 12 healthcare professionals (pediatricians, registered dietitians, public health nurses, nurses, epidemiologists, childcare workers, and kindergarten teachers). Each item was assessed using three criteria: (1) relevance (whether the question is appropriate for screening preschoolers’ nutritional risk); (2) clarity (whether the question is clearly stated); and (3) simplicity (whether the question is simple, easy to understand, and appropriate for parents to answer). Each criterion was rated on a 4-point Likert scale, with higher scores indicating greater validity. The content validity index (CVI) was determined as the proportion of evaluators who rated an item ≥ 3. A CVI ≥ 0.79 was considered acceptable, 0.70–0.78 required modification, and ≤0.69 led to item removal [23].

Next, a focus group interview (FGI) was conducted with professionals from educational and child welfare institutions, as well as parents, to assess face validity. Using the general public health literacy scale developed by Ishikawa et al. [24], the criterion-related validity was examined. Based on the median health literacy score, the participants were stratified into two groups to compare their questionnaire scores.

For reliability testing, a test–retest method was applied, the survey was administered twice with a 2-week interval, and the intraclass correlation coefficient (ICC 1, 2) was calculated. Moreover, Cronbach’s alpha and McDonald’s omega (ω) coefficient was calculated to assess internal consistency.

### 2.3. Ethical Considerations

During the online survey, an explanation of the study was displayed on the screen, and participants consented to participate by proceeding with the survey. The consent form clarified that participation was voluntary and that no personally identifiable information would be collected. This study was approved by the research ethics committees of Jissen Women’s University and Tokyo University of Agriculture (approval nos. and dates of approval: H2023-27, on 2 May 2024, and 2294, on 15 May 2024).

### 2.4. Definition of Early BMI Increase as a Proxy for Adiposity Rebound

BMI was calculated by dividing weight (kg) by height (m) squared. In this study, due to limited available time points, we adopted a proxy definition of AR as an increase in BMI between 18 and 36 months. While this approach differs from the conventional definition based on the age of BMI nadir [4], it aligns with Japanese pediatric guidelines that recognize such an increase as indicative of early AR and potential risk of later obesity [25]. Participants were categorized into AR group (n = 193) or non-AR group (n = 495) based on the presence or absence of BMI increase during this interval.

### 2.5. Statistical Analysis

As the desirable frequency of food intake varies by food type, the analysis used a scoring system for the intake frequency. Scores were assigned to staple foods (grains), main dishes (fish, meat, eggs, soy products), and side dishes (vegetables), and a total score was calculated as follows: (1) staple foods (grains: ≥5, 3–4, 2, or 1 time(s) per day, and rarely consumed = 1, 0, 2, or 3, and 4 points, respectively); (2) main dishes (fish, meat, eggs, and soy products [intake of any]: daily = 0 point, and 4–6, 1–3, and < 1 time(s) per week = 1, 2, and 4 points, respectively; and the average score for the four categories was calculated); and (3) side dishes (vegetables: ≥3, 2, and 1 time(s) per day = 0, 1, and 2 points, respectively, and rarely consumed = 4 points). The scoring method was based on NutriSTEP [22] and the Japanese Dietary Balance Guide for young children [26]. Higher scores indicated a less desirable dietary pattern. Intake by each food group was scored according to the preferred frequency of intake. An overall risk score (0–12 points) for staple foods, main dishes, and side dishes was calculated, with higher scores indicating poorer dietary habits. Health literacy scores were evaluated using the paired *t*-test.

For intergroup comparisons of the AR and non-AR groups, appropriate statistical tests were selected based on variable type and distribution, including the unpaired *t*-test, Mann–Whitney *U* test, or chi-square test. The Shapiro–Wilk test confirmed normality for most continuous variables; in cases where non-normal distributions were detected, the Mann–Whitney *U* test was applied. The total score was dichotomized at the median (high vs. low), and an unadjusted absolute risk difference (RD) was reported. The reference category was the group with the lower observed risk. To analyze the relationship between the risk scores of staple foods, main dishes, and side dishes with AR status, logistic regression analysis was conducted, with AR status as the dependent variable. The independent variables were examined in two ways. First, each food category (staple foods, main dishes, side dishes) was entered separately in the analysis. Second, the total score of all three categories was entered as a single variable. Based on data distribution, staple foods and side dishes were categorized into two groups (0 points = 0, ≥1 point = 1); main dishes were divided into two categories using the median value (≤1.75 and ≥2.0). Log-transformed values for total risk scores for staple foods, main dishes, and side dishes were entered as continuous variables. Model 1 included the child’s sex and age as covariates, whereas Model 2 included all variables from Model 1 plus gestational age, birth weight, daycare use, maternal employment status, maternal obesity (0,1), and paternal obesity (0,1). Statistical analyses were conducted using IBM SPSS Statistics v.29, with a significance level set at 5%.

## 3. Results

The CVI of the survey ranged from 0.83 to 1.00. The FGI-derived feedback regarding the clarity of wording was incorporated, and the revised version of the questionnaire was used in the main study. The median health literacy score was 3.6; the survey scores for the two groups, those with a health literacy score ≤ 3.6 vs. >3.6, were 21.4 ± 7.5 and 18.8 ± 6.6, respectively (*p* < 0.001). The ICC (1, 2) was 0.889 (95% CI: 0.869–0.906). The internal consistency of the dietary questionnaire was acceptable. For all 22 items, the Cronbach’s α was 0.69 and McDonald’s ω was 0.69. When limited to the three balanced-meal components (staple foods, main dishes, and side dishes), both the Cronbach’s α and McDonald’s ω were 0.68.

**Figure 1:**
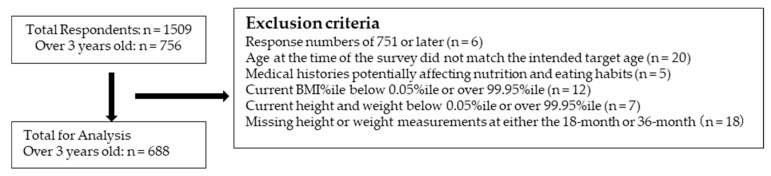
Flowchart of participants.

Among children ≥3 years, participants with response numbers ≥ 751 (n = 6), whose age at the time of the survey did not match the intended target age (n = 20), with medical histories potentially affecting nutrition and eating habits (n = 5), with a current BMI percentile < 0.05 or >99.95 (n = 12), with a current height or weight percentile < 0.05 or >99.95 (n = 7), and missing height or weight measurements at either the 18-month or 36-month checkups (n = 18) were excluded. Thus, 688 participants were included in the final analysis (Figure 1), with 193 (28.1%) and 495 (71.9%) in the AR and non-AR groups, respectively.

Most mothers were employed, and children were primarily cared for at nursery schools or kindergartens during the daytime (Table 1). The prevalence of maternal and paternal obesity was 10.3% and 25.6%, respectively.

No significant intergroup difference was observed in weight at birth; however, at 18 months, weight and BMI were lower in the AR group (both *p* < 0.001), whereas at 36 months, the height was greater in the non-AR group, and BMI was higher in the AR group (both *p* < 0.001, Table 2). Moreover, the most recent BMI was higher in the AR group (*p* = 0.003).

Supplementary Table S1 provides the results of the 22-item survey, which assessed food-group intake frequency and lifestyle factors. The intake frequency of grains and meat was lower in the AR group; however, both groups had similar exercise frequency and economic stability.

**Figure 2:**
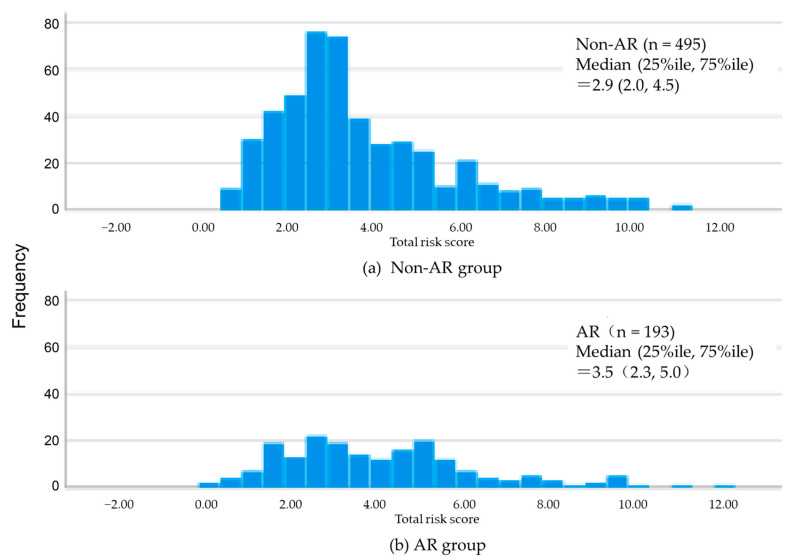
Histogram of total risk score for staple foods, main dishes, and side dishes.

The median total risk score for staple foods, main dishes, and side dishes was 2.9 in the non-AR group, with the histogram peak near the median. In contrast, the AR group had a median score of 3.5, with a second peak adjacent to 4–6 points (Figure 2). The risk scores for staple foods (*p* = 0.018) and the total risk score for staple foods, main dishes, and side dishes (*p* = 0.038) were higher in the AR group (Table 3). In contrast, no significant differences were observed in the risk scores for main dishes and side dishes.

Logistic regression analysis, with AR status as the dependent variable, showed that higher risk scores for staple foods, main dishes, and total (staple foods, main dishes, and side dishes) were associated with an increased risk of AR (Table 4). This association remained significant even after adjusting for confounding factors, such as birth weight and parental obesity (adjusted odds ratio: 2.58, 95% CI: 1.08–6.16).

In addition to odds ratios, we calculated absolute risk measures for better clinical and public health interpretability. When the total dietary risk score was dichotomized at the median, the unadjusted risk of early AR was 24.1% (89/369) in the lower-risk group and 32.6% (104/319) in the higher-risk group, yielding an absolute risk difference in percentage points of 8.5% (95% CI: 1.7 to 15.2).

## 4. Discussion

This nationwide cross-sectional analysis demonstrated that a lower frequency of balanced meals, comprising a staple food, a main dish, and a side dish, was significantly associated with early adiposity rebound (AR) among Japanese preschool children. The association remained significant after adjustment for child and parental characteristics. These findings indicate that meal balance may be an important dietary factor that is linked to early BMI rebound and subsequent obesity risk.

In this study, anthropometric data from the 18-month and 36-month health checkups that were conducted uniformly by professionals were used to determine early AR. However, the typical age of AR onset varies by region. Data from a German population indicate that the average rebound age is 5 years [27], whereas a study conducted in Chile found that approximately half of the AR cases occurred before age 5 [7]. In Japan, a longitudinal study reported that AR onset before age 4 was associated with higher BMI and risk factors for metabolic syndrome at age 12 [6]. Another Japanese study of 54,558 children observed AR at approximately 36 months in children who were overweight at age 6 [5]. A longitudinal study on Latin American children found that 18.2% of their participants experienced early AR before 3.5 years, whereas 40.0% had AR onset between 3.5 and 5 years, which indicated an earlier occurrence than in other studies [9]. In contrast, the present study found that 28.1% of children experienced AR between 1.5 and 3.0 years, which suggests a predisposition to early AR among Japanese children. Therefore, the AR calculation from anthropometric measurements recorded at 18 and 36 months in this study is appropriate.

Regarding staple food intake, 68.7% of non-AR children consumed staple foods 3–4 times a day, whereas AR children tended to consume staple foods less frequently. A similar trend was observed for main dish consumption, where AR children had lower intake frequencies. Although portion sizes were not assessed, the higher recent BMI in AR children suggests that they may be consuming larger portions in fewer meals. In a large national sample of Iranian children and adolescents aged 6–18 years, those with lower meal frequency had a significantly higher risk of overweight, obesity, and abdominal obesity compared with those consuming three meals per day [28]. In early childhood, energy intake is necessary not only for daily activities but also for growth, and therefore, the distribution of energy intake across meals is likely beneficial.

Side dishes were not independently associated with AR. Only approximately 30% of children in both groups consumed side dishes three or more times per day, whereas 23% and 28% of children in the non-AR and AR groups, respectively, consumed side dishes less than one time per day, which suggests that the overall vegetable intake was insufficient. A Japanese study that compared 3-year-olds’ intake of staple food, main dish, and side dish to recommended serving sizes found that side dishes had the lowest scores among all food categories [29]. An Australian study that investigated the recommended and actual dietary patterns in 1- to 2-year-olds reported that approximately two-thirds of children did not consume enough vegetables [30]. A meta-analysis examined whether lowering the standard for adequate fruit and vegetable intake to at least four servings per day or at least five times per week could reduce the risk of childhood obesity; however, the results showed no significant reduction in obesity risk in children [31]. Therefore, further interventional studies are needed to determine whether increased vegetable intake can help prevent AR or improve obesity.

As opposed to the examination of staple foods, main dishes, and side dishes separately, the combined risk score that we used in this study showed a significant association with AR that persisted even after adjusting for confounding factors, such as parental obesity and birth weight. Thus, a comprehensive dietary index for assessing meal combinations may provide a more accurate evaluation of nutritional balance and health outcomes than one that analyzes individual food groups.

Ishikawa-Takata et al. [32] analyzed data from the National Health and Nutrition Survey and reported that a higher frequency of meals consisting of staple foods, main dishes, and side dishes positively correlated with higher nutrient intake and improved overall dietary quality. Furthermore, a longitudinal study showed that a lower frequency of consuming meals with staple foods, main dishes, and side dishes was associated with a higher risk of weight gain over 3 years [33]. In contrast, research that focused on single nutrients and AR found no consistent relationship between habitual protein intake during childhood and AR [27,34]. This suggests that the frequency of balanced meal intake, rather than individual nutrient or food group intake, has greater predictive accuracy for overweight and obesity risk. Although previous studies [32,33] focused on adults, the present study indicates that evaluating meal balance based on staple foods, main dishes, and side dishes could be a useful indicator for AR prevention in children.

Balanced meals typically provide adequate amounts of energy, protein, and micronutrients, and thereby lead to stable appetite regulation and appropriate weight gain. Macronutrients, such as protein, increase satiety relative to carbohydrates by stimulating gut–brain hormones such as glucagon-like peptide-1 (GLP-1), cholecystokinin (CCK), and peptide YY (PYY), thereby reducing subsequent energy intake [35]. Frequent omission of any major meal component—staple food, main dish, or side dish—may disturb the equilibrium of macronutrients and lead to unstable energy balance and dysregulation of appetite-related hormones, including ghrelin and leptin, which are key mediators of food intake control and weight regulation [36]. These disruptions in early childhood may constitute mechanisms that accelerate AR. Repeated exposure to well-structured meals during early childhood may also shape food preferences and enhance self-regulation of energy intake, which is critical for long-term weight control [35]. Conversely, irregular meal composition and energy imbalance may condition children to prefer high-energy, palatable foods, reinforcing positive energy balance and promoting early AR. These physiological and behavioral pathways provide a plausible explanation for the observed association between low frequency of balanced meals and early AR.

In addition to hormonal regulation, emerging evidence suggests that gut microbiota may play a mediating role. An obesity-associated microbiome, characterized by reduced diversity and an increased abundance of Firmicutes with a concomitant reduction in Bacteroidetes, has been shown to enhance the capacity for energy harvest from the diet [37]. In a seminal experiment, Turnbaugh et al. demonstrated that germ-free mice colonized with microbiota from obese donors accumulated significantly more body fat than those colonized with lean microbiota, despite identical food intake [37]. Such findings indicate that dysbiosis not only alters energy extraction but also promotes low-grade inflammation and metabolic dysregulation, thereby contributing to the risk of obesity. High-fat diets, in particular, have been shown to modify gut microbial composition and energy harvesting efficiency [38], while diets rich in fiber promote the production of short-chain fatty acids, which play a central role in appetite regulation and host energy metabolism [39]. Thus, both hormonal dysregulation and microbiota-mediated mechanisms may underlie the observed association between lower frequency of balanced meals and early AR.

The finding that AR, a known risk factor for future obesity, is associated with the simplified total risk score of staple foods, main dishes, and side dishes has important implications for early nutritional assessment and intervention. Infant and toddler health checkups provide a unique opportunity to assess growth trajectories and involve multidisciplinary professionals such as physicians, public health nurses, and registered dietitians. Integrating diet-based nutritional assessments into routine screenings could enhance the early detection of nutritional risks. Although parents have limited opportunities to interact with professionals, using health checkup data to provide timely nutritional guidance with a simple and actionable message that emphasizes a balanced intake of staple foods, main dishes, and side dishes represents a potential strategy to help mitigate the risk of childhood obesity. Collectively, our findings support the importance of prioritizing comprehensive dietary assessment over individual food-group evaluations in early childhood health policies. This study offers a novel contribution by proposing balanced-meal frequency as a feasible screening measure, while also recognizing the need for prospective studies with larger sample sizes to confirm these associations and further establish its predictive validity.

### Strengths and Limitations

The strengths of this study include the large nationwide sample, the use of the Maternal and Child Health Handbook for reliable anthropometric data, and the utilization of a validated 22-item Japanese Nutrition Screening Questionnaire. The primary limitation of this study is the cross-sectional and secondary design, which does not allow causal inference. Another limitation is this non-prospective design, as it examined the association of current dietary habits with past AR based on BMI change from 18 months to 3 years. Ideally, the relationship between dietary habits at 18 months and subsequent obesity should be assessed; however, the lack of dietary data at 18 months in our study precluded this approach. This lack of a forward time sequence precluded the detection of a causal relationship between dietary habits and AR. Further prospective studies are needed to determine whether consuming a balanced diet of staple foods, main dishes, and side dishes can prevent AR. Furthermore, the survey used in this study did not account for portion sizes or quantitative intake data, and assessed only the consumption frequency. Although this reflects the survey’s intended use as a simple nutritional screening tool, it introduces limitations because individual meal portions likely vary. Thus, accurately capturing children’s actual food intake remains a challenge. Further research is needed to validate whether the risk score for staple foods, main dishes, and side dishes used in this study correlates with actual serving sizes and overall nutritional balance, which could be examined through dietary record-based studies. Furthermore, this study was conducted through an internet-based survey, allowing nationwide data collection. However, the recruitment of participants from an online panel may limit the socioeconomic and geographic representativeness of the sample. Selection bias may be present, as survey participants may have a higher interest in health-related information, leading to higher health literacy than the general population. Additionally, if a significant number of participants completed the survey for financial incentives, measurement errors could have affected the results. Another limitation is that stratified analyses to examine potential effect modifications by factors such as the child’s sex or parental obesity were not conducted. Although such analyses are important, we chose not to perform them in the present study in order to maintain statistical power in the main analyses and to avoid the complexity associated with multiple testing corrections. Finally, the relatively small sample for a nationwide study may limit the generalizability of the findings. Future studies with larger and more diverse cohorts are needed to confirm these associations.

## 5. Conclusions

A lower frequency of balanced meals was found to be associated with early AR in this cross-sectional sample. Our findings support the further investigation of interventions promoting balanced meal patterns as a potential component of public health programs to delay AR and reduce the long-term obesity risk.

## Figures and Tables

**Table 1:** Clinicodemographic characteristics of the participants.

	Total (n = 688)		Adiposity Rebound *			
			No (n = 495)		Yes (n = 193)	
Sex of the child	341	(49.6)	264	(53.3)	77	(39.9)
	347	(50.4)	231	(46.7)	116	(60.1)
Age of the child	4.6	±0.8	4.6	±0.8	4.6	±0.9
Age of the mother	36.0	±5.0	36.0	±4.9	35.8	±5.1
Employment status of the mother	495	(71.9)	353	(71.3)	142	(73.6)
Primary Daytime Childcare Providers (multiple answers)						
Nursery School	284	(41.3)	205	(41.4)	79	(40.9)
Kindergarten	269	(39.1)	191	(38.6)	78	(40.4)
Certified Childcare Center ^#^	130	(18.9)	91	(18.4)	39	(20.2)
Grandparents/ Relatives	3	(0.4)	1	(0.2)	2	(1.0)
Other	13	(1.9)	11	(2.2)	2	(1.0)
Mother’s BMI (kg/m^2^)	21.2	±3.3	21.3	±3.3	21.0	±3.1
Maternal Obesity (BMI ≥ 25 kg/m^2^)	71	(10.3)	51	(10.3)	20	(10.4)
Father’s BMI (kg/m^2^)	23.3	±3.3	23.3	±3.4	23.1	±3.2
Paternal Obesity (BMI ≥ 25 kg/m^2^)	176	(25.6)	128	(25.9)	48	(24.9)

n (%) or mean ± SD. * Difference in BMI at the 36-month health checkup and the 18-month health checkup. ^#^ A facility that combines the functions of both a kindergarten and a nursery school that provides early childhood education and care for preschoolers.

**Table 2:** Association of AR * and Body-related Factors.

	Adiposity Rebound *				*p*
	No (n = 495)		Yes (n = 193)		
Gestational age ^#^	38.7	±2.4	38.7	±1.9	0.540
Weight at birth ^#^	2998	±415	3011	±447	0.698
18 months					
Height (cm) ^#^	80.3	±3.0	80.5	±3.1	0.759
Weight (kg) ^#^	10.7	±1.1	10.1	±1.0	<0.001
BMI (kg/m^2^) ^#^	16.5	±1.2	15.5	±1.1	<0.001
36 months					
Height (cm) ^§^	94.9	±3.9	93.7	±3.7	<0.001
Weight (kg) ^#^	14.1	±1.5	14.1	±1.5	0.426
BMI (kg/m^2^) ^#^	15.6	±1.1	16.1	±1.1	<0.001
Most Recent Measurements					
Height (cm) ^#^	103.1	±6.8	102.2	±7.8	0.191
Weight (kg) ^#^	16.5	±2.4	16.5	±2.8	0.993
BMI (kg/m^2^)	15.5	±1.3	15.8	±1.2	0.003

Mean ± SD. * Difference in BMI at the 36-month health checkup and the 18-month health checkup. ^#^ Mann–Whitney *U* test. ^§^ Unpaired *t*-test.

**Table 3:** Association of AR* with the risk scores for staple foods, main dishes, and side dishes ^†^.

	Total (n = 688)		Adiposity Rebound *				*p*
			No (n = 495)		Yes (n = 193)		
Staple Foods ^#^	0.7	±1.1	0.6	±1.1	0.8	±1.1	0.018
Main Dishes ^§^	1.7	±0.7	1.7	±0.7	1.8	±0.8	0.097
Side Dishes ^#^	1.2	±1.2	1.1	±1.1	1.2	±1.2	0.765
Total (Staple Foods, Main Dishes, Side Dishes) ^§,‡^	3.6	±2.2	3.5	±2.1	3.9	±2.3	0.038

Mean ± SD. * AR group = children with >0 difference in BMI at 18 and 36 months. ^†^ A higher score indicates a higher risk. ^#^ Mann–Whitney *U* test. ^§^ Unpaired *t*-test. ^‡^ The total score for staple foods (grains), main dishes (fish, meat, egg, soy products), and side dishes (vegetables).

**Table 4:** Logistic regression analysis with AR as the dependent variable.

Risk Score	Model 1			Model 2		
	Exp (B)	95% CI	*p*	Exp (B)	95% CI	*p*
Staple Foods	1.48	(1.04–2.09)	0.029	1.48	(1.03–2.12)	0.034
Main Dishes	1.42	(1.01–2.00)	0.043	1.44	(1.02–2.04)	0.040
Side Dishes	1.30	(0.89–1.90)	0.177	1.33	(0.90–1.97)	0.148
Total (Staple Foods, Main Dishes, Side Dishes) ^‡^	2.19	(0.94–5.10)	0.068	2.58	(1.08–6.16)	0.033

Dependent Variable: Presence or absence of AR. Independent Variable: Staple Foods/Side Dishes (score: 0, 1+), Main Dishes (≤1.75 and ≥2.0), Total Risk Score (log-transformed continuous variable). Model 1: Child’s age and sex were used as covariates. Model 2: Child’s age, sex, gestational age, weight at birth, daycare use, mother’s employment status, mother’s obesity (0, 1), and paternal obesity (0, 1) were used as covariates. ^‡^ Total score for staple foods (grains), main dishes (mean score for fish, meat, egg, soy products), and side dishes (vegetables).

## Data Availability

The data sets used and/or analyzed during the current study are available from the corresponding author on reasonable request.

## Supplementary Materials

The following supporting information can be downloaded at: https://www.mdpi.com/article/10.3390/nu17193183/s1, Table S1: Association of AR with food intake frequency by each food group, and of AR with lifestyle (simple tabulation).

## Abbreviations

The following abbreviations are used in this manuscript:

BMI	body mass index
BAZ	BMI for age z-score
WAZ	weight-for-age z-score
WHO	World Health Organization
AR	Adiposity Rebound
COVID-19	coronavirus disease
CVI	content validity index
FGI	focus group interview
